# Analysis of interactions between the epigenome and structural mutability of the genome using Genboree workbench tools

**DOI:** 10.1186/1471-2105-15-S7-S2

**Published:** 2014-05-28

**Authors:** Cristian Coarfa, Christina Stewart Pichot, Andrew Jackson, Arpit Tandon, Viren Amin, Sriram Raghuraman, Sameer Paithankar, Adrian V Lee, Sean E McGuire, Aleksandar Milosavljevic

**Affiliations:** 1 Molecular & Cellular Biology, Baylor College of Medicine, Houston, TX 77030, USA; 2 Molecular & Human Genetics, Baylor College of Medicine, Houston, TX 77030, USA; 3 Social and Scientific Systems, Durham, NC 27713, USA; 4 Pharmacology and Chemical Biology, University of Pittsburgh, Pittsburgh, PA 15213, USA; 5 Department of Radiation Oncology, University of Texas MD Anderson Cancer Center, Houston TX 77030, USA

## Abstract

**Background:**

Interactions between the epigenome and structural genomic variation are potentially bi-directional. In one direction, structural variants may cause epigenomic changes in *cis*. In the other direction, specific local epigenomic states such as DNA hypomethylation associate with local genomic instability.

**Methods:**

To study these interactions, we have developed several tools and exposed them to the scientific community using the Software-as-a-Service model via the Genboree Workbench. One key tool is Breakout, an algorithm for fast and accurate detection of structural variants from mate pair sequencing data.

**Results:**

By applying Breakout and other Genboree Workbench tools we map breakpoints in breast and prostate cancer cell lines and tumors, discriminate between polymorphic breakpoints of germline origin and those of somatic origin, and analyze both types of breakpoints in the context of the Human Epigenome Atlas, ENCODE databases, and other sources of epigenomic profiles. We confirm previous findings that genomic instability in human germline associates with hypomethylation of DNA, binding sites of Suz12, a key member of the PRC2 Polycomb complex, and with PRC2-associated histone marks H3K27me3 and H3K9me3. Breakpoints in germline and in breast cancer associate with distal regulatory of active gene transcription. Breast cancer cell lines and tumors show distinct patterns of structural mutability depending on their ER, PR, or HER2 status.

**Conclusions:**

The patterns of association that we detected suggest that cell-type specific epigenomes may determine cell-type specific patterns of selective structural mutability of the genome.

## Background

Historically, the first link ever discovered between chromatin structure and epigenetics was due to a structural genomic variant - a breakpoint induced by a chromosomal inversion on the × chromosome in *Drosophila *[1]. This variant explained position-effect variegation of the *Drosophila *eye color by stochastic spreading of heterochromation across an inversion-induced breakpoint and stochastic silencing of the *Drosophila *eye color gene [1]. We now also know that interactions between the genome and the epigenome may be bi-directional, one direction being exemplified by position-effect variegation of *Drosophila's *eye color, and the other direction suggested by our recent discovery that hypomethylation of genomic DNA in human germline associates with local genomic instability [2].

The opportunity to gain insights into epigenome-genome interactions is fast emerging. Structural genomic variants, including inversions, duplications, deletions, and translocations are being mapped on large scale in human germline and in cancer using mate-pair sequencing. A number of informatics challenges are yet to be addressed before this sequencing data becomes useful for analysis in the context of the epigenome. The detection algorithms must achieve sufficient accuracy to enable genome-scale correlation of chromosomal breakpoints with epigenomic features even without costly validation experiments. The structural aberrations should be interpretable in the context of rapidly growing public databases of transcription factor binding sites and epigenomic profiles. One practical challenge is the deployment of multiple analysis tools and databases with reproducible and transparent records of analyses.

To address these challenges we developed a structural variant analysis toolset, the Structural Variants Analysis Toolset, and deployed it using the Genboree Workbench, a collaborative environment for integrative analysis of genomic and epigenomic data. A key tool within the toolset is Breakout, a parallel algorithm for breakpoint calling from mate-pair reads that, when compared to state-of-the-art tools, achieves superior sensitivity and specificity. Breakout retains sensitivity even for low read coverage and is therefore suitable for profiling samples where only a small fraction of cells carry specific aberrations such as tumor samples with low tumor cell purity. Additional tools within the toolset enable integrative analysis using structural variation databases, such as the one generated by the 1000 Genomes Project [3-5], and other genomic and epigenomic databases required for integrative interpretation of breakpoint data, including ENCODE [6,7] and the Human Epigenome Atlas produced by the NIH Epigenomic Roadmap Project [8].

We herein apply the Genboree Workbench tools to address a number of questions regarding the interactions of the epigenome with structural variation of the genome. We examine those interactions in both directions. In one direction, we identify structural genomic variants in cancer and their association with epigenomic changes in *cis*. In the other direction, we validate and then extend our recent discovery that hypomethylation of genomic DNA in human germline associates with local genomic instability [2]. We validate the association at high resolution, using mate-pair sequencing data, by performing an extensive meta-analysis using ChIP-Seq data and epigenomic marks from ENCODE, Cistrome and other projects. Finally, we expand our analysis beyond germline to genomic instability in breast cancer and discover cell-type specific associations between the epigenome and selective structural mutability.

## Methods

### Breakpoint calling using Breakout

To discover breakpoints in tumor samples using long insert (4-6 Kbp) mate pairs, available widely on platforms such as SOLiD, Illumina, or 454, we developed Breakout, a novel chromosomal breakpoints detection algorithm and software package. To harness the parallelism of multicore processors, Breakout decomposes analysis steps into balanced segments that can be processed independently. The input consists of SAM/BAM [9] files containing the uniquely mapped mate pairs, as produced by mapping programs such as BFAST [10], bwa [11,12], or Pash [13]. Breakout calculates the distribution of insert sizes for all mate pairs with both ends mapped on the same chromosome in expected strand orientation. The consistent mate pair range I_min _to I_max _is calculated to capture the 0.5-99.5 percentile range of the distribution. A user can override these bounds and select a custom range instead.

Breakout splits mappings of forward and reverse reads into balanced groups. For each read group, it hashes forward read mappings, and then streams over the reverse read mappings. It separates the mate pairs into two classes: consistent mate pairs, defined as those with read mappings on the same chromosome, with the expected relative strand orientation, and within the insert size range (I_min_, I_max_), and inconsistent mate pairs.

**Figure 1:**
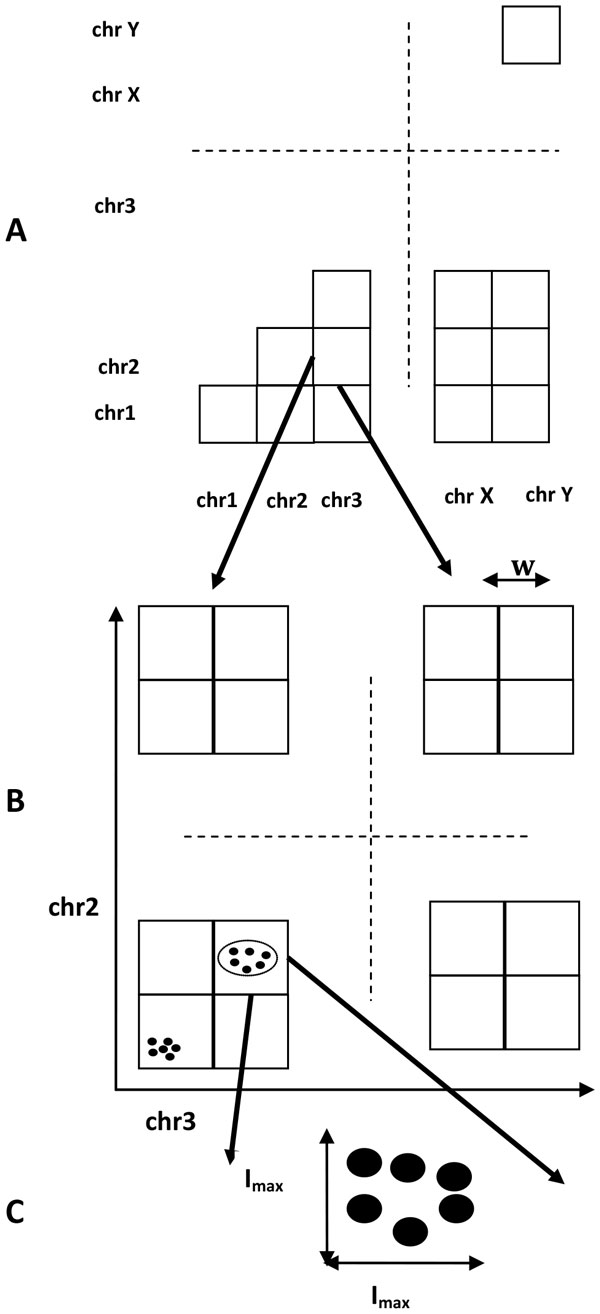
**Breakout iterative grid clustering strategy**. Breakout employs iterative grid clustering of mate pairs in three steps: (A) Identifies read pairs mapped on the same pair of chromosomes. (B) For each chromosome pair, it performs coarse-level greedy clustering. (C) It refines mate pair clusters. The example shows detection of a breakpoint between chromosomes 2 and 3.

The inconsistent mate pairs are then examined to identify those arising from the same physical breakpoint, by clustering them based on the proximity of their end-reads. To take advantage of the parallelism inherent in modern CPUs, Breakout applies a grid decomposition iterative approach to mate pair clustering, presented in Figure 1. First, it splits mate pairs by the pair of chromosomes they connect (Figure 1A). For both inter- and intra-chromosomal inconsistent mate pairs, Breakout considers a chromosome-tiling fixed size sliding window w, with w exceeding the insert size I_max_. Mate pairs are sorted in increasing order of the genomic window index and are clustered using a greedy heuristic (Figure 1B). A hierarchical clustering step processes mate pairs in hotspots of rearrangement and further refines the detected breakpoints (Figure 1C). Breakout accounts for clonal copies by keeping only one representative mate pair for each set of identical copies. To account for sequencing bias, Breakout requires that within each cluster read ends be at least 50 bp apart.

Integrating breakpoint information from multiple samples in the context of known structural polymorphisms and other genomic information.

When mapping breakpoints in cancer cells it is of interest to identify the breakpoints that are likely of germline origin by comparing against the breakpoints detected using the mate-pairs from the 1000 Genomes project and other databases of structural polymorphisms. Genboree Workbench enables such integration via a collection of tools.

The ***Collect Insert Size ***tool quantifies the distribution of the mate pair insert size, and suggests to the user a range for lower and upper insert size bounds corresponding to the 0.5-99.5 percentiles of the cumulative insert size distribution.

The ***Intersect SVs ***tool performs elementary set operations of intersection and difference on Breakout outputs. Overlapping breakpoints are considered identical for the purpose of these set operations and are defined as follows. Let breakpoint B_1 _have the coordinates chrA:[b_1_,e_1_]-chrB[b_2_,e_2_] and the breakpoint B_2 _have the coordinates chrA:[b_1_',e_1_']-chrB:[b_2_',e_2_']. Let I_max _be the maximum insert size for the two experiments. Breakpoints B_1 _and B_2 _overlap if min(|b_1_-b_1_'|,|e_1_-e_1_'|,|b_1_-e_1_'|,|e_1_-b_1_'|) <=I_max _and min(|b_2_-b_2_'|,|e_2_-e_2_'|,|b_2_-e_2_'|,|e_2_-b_2_'|) <=I_max_.

The ***Report Multiple SVs ***tool calculates overlaps among breakpoint sets from multiple samples or between breakpoints and or other genomic features. For each input set, the tool reports at breakpoint level overlaps with breakpoints from other input sets, with RefSeq genes, and even with custom genomic features uploaded by a user. This is useful for distinguishing polymorphic germline breakpoints from breakpoints detected in cancer genomes. Whereas some tumor samples are sequenced in parallel with normal controls, many patient samples and most cell lines have no available germline reference. We computed breakpoints based on long insert mate pairs datasets from the 1000 Genomes project database [4], and by running the Report Multiple SVs tool we could identify overlapping breakpoints and thus separate the breakpoints detected in cancer samples into two groups - the overlapping ones that are likely of germline origin and the non-overlapping ones that are putatively of somatic origin.

#### Integrative analysis of epigenomic features and breakpoints

The amount of epigenomic data that is becoming publicly available is rapidly growing [14-18], making it possible to explore epigenomic correlates of chromosomal aberrations on a large scale. One specific epigenomic data set is the Human Epigenome Atlas (http://www.epigenomeatlas.org), generated by the NIH Epigenomic Roadmap Initiative.

To enable a basic exploration of the epigenomic correlates of breakpoints, we developed the Epigenomic Enrichment tool, which determines enrichment of epigenomic features in the vicinity of breakpoints. In some use cases, the enrichment may suggest that the epigenomic features cause genomic instability. The inputs are structural variation tracks and the tracks containing discrete epigenomic features such as histone modification peaks, areas of low/intermediate/high methylation. Enrichment values are calculated separately for each class of variant: deletions, insertions, inversions, and translocations. In the first step of the algorithm, epigenomic features within a user-defined window (50,000 bp default) surrounding the breakpoints are identified. Next, a permutation test determines the number of features expected to occur by chance around breakpoints and reports the average enrichment and the associated p-value.

#### Integration of the tools within the Genboree Workbench

**Figure 2:**
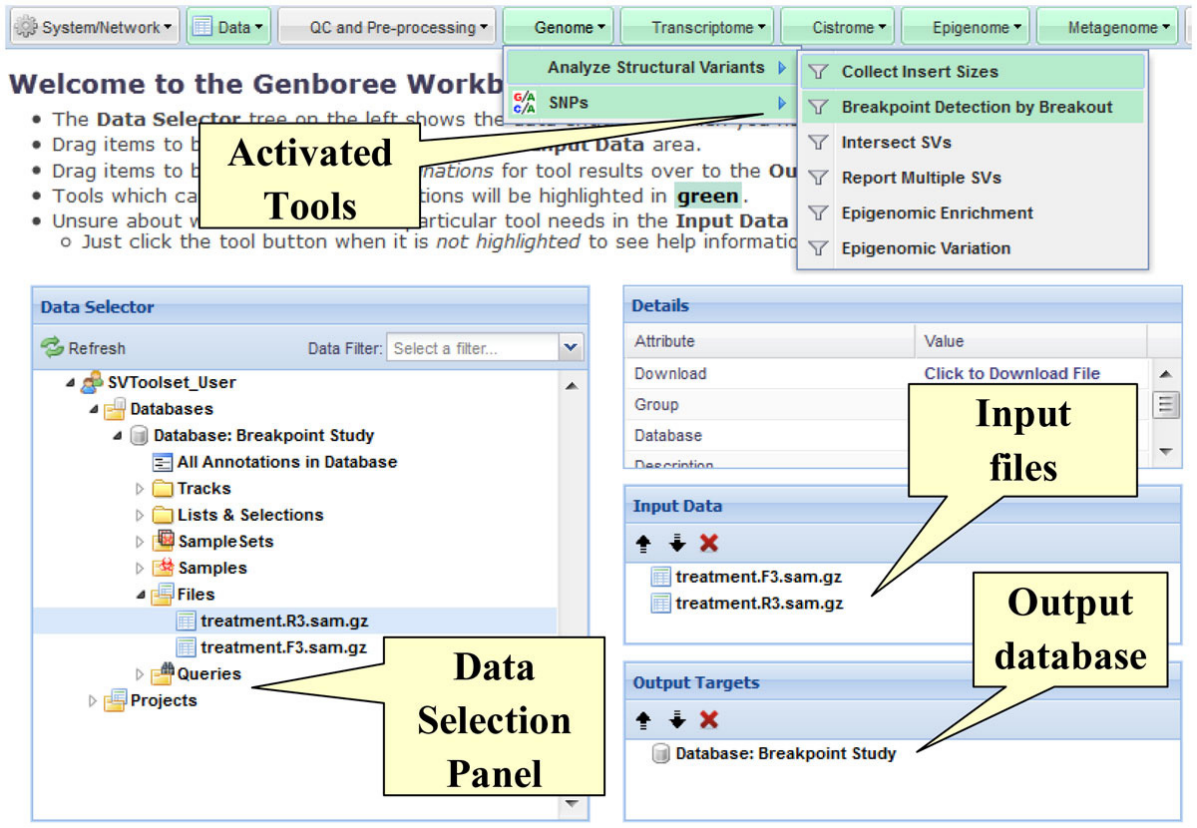
**Genboree Workbench interface**. A user can explore a data tree containing various data types: sequencing results, tool results, in multiple formats, via a data selection panel. The user drags files to be used as tool inputs into the Input Data panel, and an output databases into the Output Targets panel. Tools which can run on the types of inputs and outputs selected are highlighted in the menu and are ready for launch using either default or user-specified parameters.

The tools described in the previous section are available online within the Genboree Workbench (Figure 2). Genboree provides web-based services for groups of researchers to share, visualize, and analyze genomic annotations and raw data files. The Workbench is implemented using Genboree REST APIs [19]which are used to retrieve appropriate tool configuration dialogs, transfer data, and submit configured tool jobs. The Genboree API server queues a job for execution on a compute cluster; following execution, a notification email is sent to the researcher containing success (or failure) information, a summary of key results, and links to any additional information and visualizations. The raw result files and visualization images are available for download from the Workbench itself in the "Files" area of the selected output database. Users can configure and submit tools directly via the API, enabling a scripted interaction or integration into local analysis pipelines.

The Genboree Workbench was designed around a core of robust principles. Tools are exposed to users via a uniform graphical interface: the user is presented with a data tree, a data object details panel, an input panel, and an output panel (Figure 2). Tools are available in the cascading menu at the top of the page. The data tree displays the natural hierarchy of object within Genboree: user groups have one or more databases for holding the data and within a database are several types of entities--including data files such as FASTA sequence files, SAM/BAM files, Excel spreadsheets with clinical metadata, results of previous tool runs, etc. When selected in the data selection tree, the details of the object are displayed in a dedicated panel. The researcher drags-and-drops input data from the tree into the Input Data panel, and drags a suitable database in which to store the tool results into the Output Targets panel. Tools whose input and output criteria have been met are highlighted in the tool menu which, when clicked, cause the appropriate dialog to appear for reviewing and customizing tool-specific parameters. Wherever possible, Workbench tool dialogs will have sensible default parameters defined to handle the most common scenarios. Each tool has a help dialog describing each parameter, as well as the input and output panel criteria for the tool.

Some tools operate on the result files or entire result folders of other tools, and file format conversion are handled automatically. In all cases, the user is informed whether the tool job has been accepted, rejected (and why), or if they first need to confirm a warning condition. Tools are queued for execution along with jobs submitted by other users and generally run on a first-come, first-serve basis. The Workbench is also the primary means for sharing tool results with collaborators and downloading the result file(s), which are generally compressed to save storage space and network bandwidth. The Genboree Workbench can be accessed at http://genboree.org/java-bin/workbench.jsp, using browsers such as Internet Explorer, version 8 and higher, and Mozilla Firefox, version 6 and higher. A tutorial and sample datasets are available at http://genboree.org/breakpoints.

Breakout and the epigenome enrichment analysis tools can be downloaded at http://www.brl.bcm.tmc.edu/breakout/breakoutDownload.rhtml.

## Results

### Breakout exhibits superior performance on low-coverage breakpoint detection

To compare Breakout with other structural variation analysis tools, we used as a benchmark structural variants in the HCC1954 breast cancer cell line. HCC1954 was previously characterized using high-coverage mapping with short insert size pairs and high stringency validation by Stephens *et al *[20]. The set of 244 previously reported somatic variants [20] were used as a positive control. An HCC1954 dataset was sequenced using the SOLiD, yielding 72 million mate pairs; the reads were mapped onto the human genome, NCBI Build 36/UCSC hg18 using bfast [10].

Breakout was compared with VariationHunter [21] and GASV [22]. VariationHunter [21] models the structural variation discovery using the set cover problem, is optimized for accurate detection of variants in normal genomes, and performs rigorous filtering of false positives. GASV[22] models the structural variation as a two-dimensional geometrical intersection of polygonal areas. GASV has been used to analyze both normal and cancer genomes. To facilitate comparison with Variation Hunter, which detects only intra-chromosomal breakpoints, we performed calling of structural variants twice, once for the complete set of inter- and intra-chromosomal events, and then second time for the subset of intra-chromosomal events only. For all three callers the minimum variant calling threshold was set to three reads per reported structural variant. To evaluate the sensitivity of the structural variation detectors, we first determined the number of benchmark somatic variants with at least one supporting read in the SOLID sequencing set, effectively discovering an upper bound for the overlap with the published set of somatic structural variants. There were 124 intra-chromosomal variants with one supporting read and 179 overall variants with one supporting read, as shown in Table 1. At three reads per variant, Breakout detects 75% of the intrachromosomal events and 75% of the overall structural variants, VariationHunter detects 9% of the intra-chromosomal variants, and GASV detects 66% of the intra-chromosomal somatic variants but only 50% of the overall of somatic variants.

**Table 1:** Overlap with previously published and validated set of 244 somatic variants in HCC1954.

Experiment		Events	Somatic
Intra- chromosomal breakpoints	*1 read pair*	*395,290*	*124*
	
	VariationHunter, 3 read pairs	508	11 (9%)
	
	Breakout, 3 read pairs	1,184	93 (75%)
	
	GASV, 3 read pairs	1,714	82 (66%)

All breakpoints	*1 read pair *	*5,773,399*	*179*
	
	Breakout, 3 read pairs	1,377	135 (75%)
	
	GASV, 3 read pairs	5,565	90 (50%)

The numbers in the parentheses indicate percentage of detected breakpoints relative to the maximum number of detectable breakpoints (overlapping with at least one read pair).

**Figure 3:**
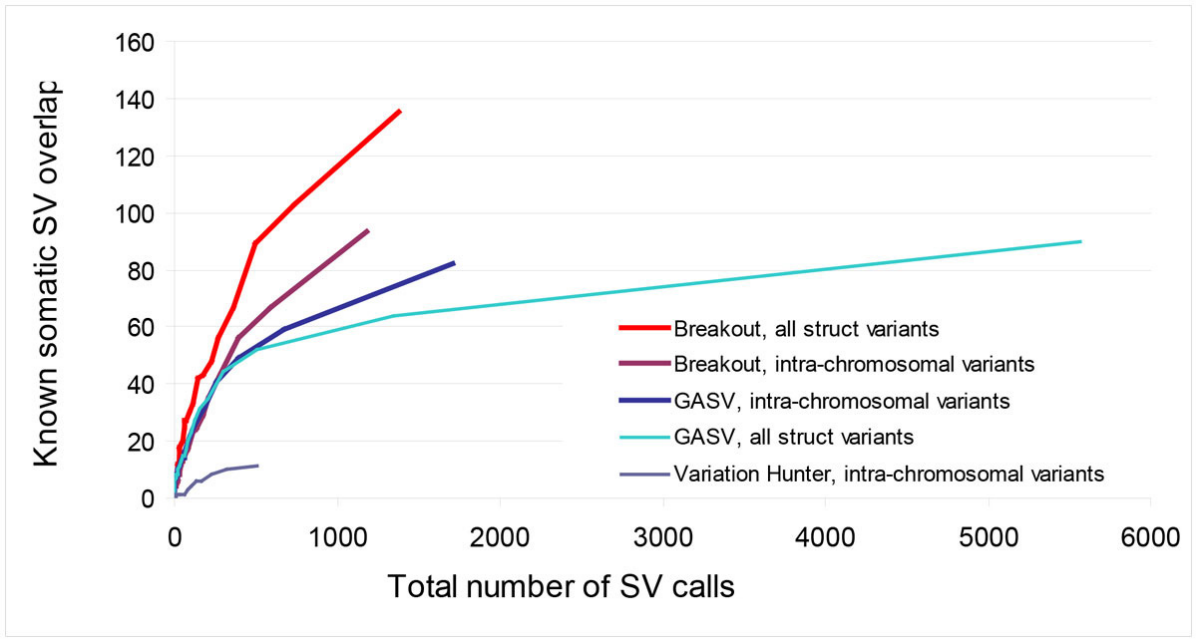
**ROC-type curves for Breakout, VariationHunter, and GASV on the HCC1954 benchmark**. Variation Hunter, optimized for normal genomes, lacks sensitivity for cancer genomes. For structural variants with high read coverage Breakout and GASV achieve similar performance. Breakout outperforms GASV with respect to sensitivity and specificity for somatic structural variants with low read coverage

By varying the minimum number of supporting mate pairs per structural variant, we derived Receiver Operator Characteristic (ROC)-type curves for Breakout, VariationHunter, and GASV, illustrated in Figure 3. This comparison methodology, used previously in other studies [21,23], uses the total number of structural variant calls as a surrogate for the unknown false positive detection rate. Variation Hunter, being designed for normal genomes, is extremely conservative and therefore not suitable for analysis of cancer genomes. Breakout and GASV achieve similar performance on somatic structural variants with high read coverage. Breakout outperforms GASV with respect to sensitivity and specificity for somatic structural variants with low read coverage, particularly for inter-chromosomal translocations. Sensitive detection at low physical coverage of breakpoints is critical when analyzing heterogeneous samples where only a fraction of cells within the sample may carry a breakpoint.

#### The Genboree Toolset accurately detects genes affected by putatively somatic mutations in the PC-3 prostate cancer cell line

To validate our integrative analysis toolset, we analyzed structural variation in PC-3, a metastatic prostate cancer cell line, and PrEC, a non-tumorigenic epithelial prostate cell line. The two cell lines were sequenced using SOLiD mate pair sequencing, at clone coverage of 8.5x for PC-3 and 10.1x for PrEC, and mapped to NCBI Build 37/hg19 using Bfast. Breakout identified a total of 382 breakpoints in PC-3 and 1184 in PrEC, using a minimum threshold of at least 3 mate pairs per breakpoint.

**Figure 4:**
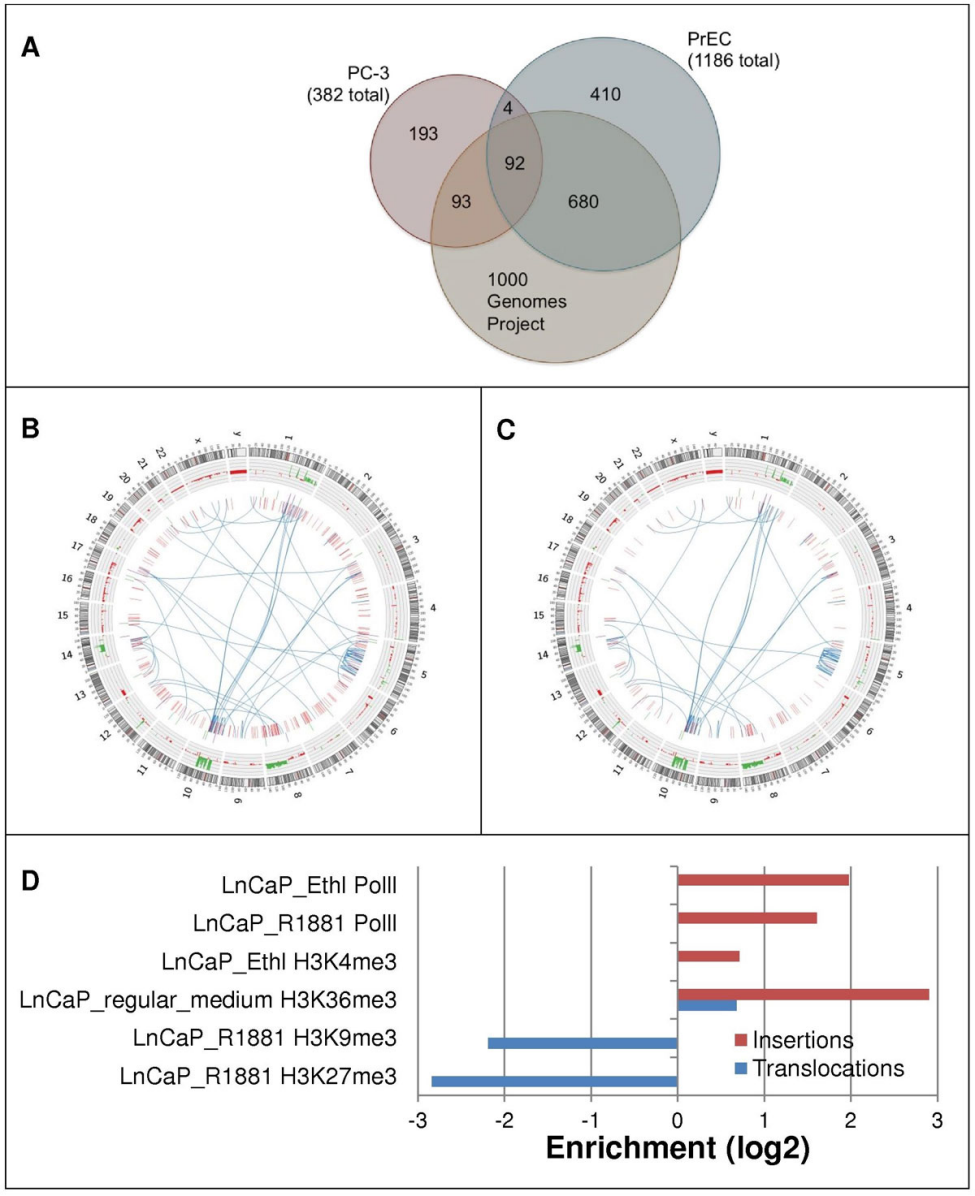
**Breakpoint analysis for the PC-3 prostate cancer cells**. (A). Breakdown of the breakpoints detected for PC-3 and PrEC: 48% of the PC-3 breakpoints and 65% of the PrEC breakpoints overlap with the breakpoints called in the 1000 Genomes Project data; 25% of the PC-3 breakpoints are common with the PrEC breakpoints. (B) Circos Plot of the PC-3 breakpoints before the subtraction of the PrEC and the 1000 Genomes Project breakpoints. (C) Circos Plot of the PC-3 breakpoints after the subtraction of the PrEC and the 1000 Genomes Project breakpoints. (D) Enrichment of epigenomic features nearby PC-3 breakpoints.

Some of the breakpoints detected in the cell lines were not due to somatic mutations but due to structural polymorphisms. In the absence of normal controls, we employed as a surrogate the mate-pairs generated by the 1000 Genomes Project [4]. Breakout was applied to call breakpoints for 50 samples in the 1000 Genomes Project database sequenced using SOLiD mate pair technology [4]. Breakout called a total of 279,256 breakpoints with at least 3 read pairs spanning each breakpoint. Using the Report Multiple SVs tool, these breakpoints were then intersected with the two sets of breakpoints in the cancer cell lines. As illustrated in Figure 4A, approximately 48% of the PC-3 breakpoints and 65% of the PrEC breakpoint are present in the normal population; 25% of the PC-3 breakpoints are present in PrEC, most of which (96%, 92 out of 96) are also present in the subset of 1000 Genomes data. Circos plots of structural variants in the PC-3 cell line are presented in Figures 4B (all 382 breakpoints) and 4C (193 breakpoints unique to PC-3 and thus putatively somatic).

We further determined breakpoints that might affect a RefSeq gene by occurring within 2kb of a gene. A subset of these breakpoints not present in PrEC or the 1000 genomes dataset were validated using PCR sequencing. The PCR primers for cross-breakpoint PCR were designed using the basic pipeline described in our earlier breast cancer study [24]. The overall PCR validation rate for translocations was 80% (8 out of 10). For a subset of structural variants with potential gene fusions, PCR primers and conditions were further optimized, leading to a validation rate of 83% (5 out of 6). The set of 193 structural variants unique to PC-3 was used to nominate 202 potentially affected genes for further study, including known translocated oncogenes MSI2 and *RAD51L1*(*RAD51B*) [25].

#### The Genboree Toolset detects epigenomically mediated regulation of genes affected by somatic aberrations

The set of 193 putatively somatic structural variants unique to PC-3 and the 202 potentially affected genes were next analyzed for enrichment of TF binding sites and other epigenomic features. Gene ontology and pathway analysis (GSEA) [26,27] revealed a significant enrichment in genes with promoter regions containing a progesterone receptor motif (p = 5.95 × 10^-5^; GSEA motif V$PR_01 [28]), as well as for homeobox gene *MEIS1 *(p = 1.12 × 10^-9^), *ESRRA *(estrogen-related receptor alpha, p = 3.47 × 10^-4^), and *FOXA1 *(p = 7.82 × 10^-4^).

Using the *Epigenomic Enrichment *tool, we quantified the enrichments of a large set of epigenomic features determined for the LnCAP prostate cancer cell line [29] around the putative somatic breakpoints from our PC-3 dataset. Significant enrichment was discovered (p < 0.05) for the active chromatin mark H3K4me3 and for PolII around insertions, for the transcription elongation mark H3K36me3 around both insertions and translocations; loss of the repressive marks H3K9me3 and H3K27me3 was discovered around translocations, as shown in Figure 4D.

### Breakpoints present in human germline strongly associate with genomic hypomethylation

We have recently shown that hypomethylation of genomic DNA in human germline marks unstable regions in the human genome instability [2]. We set out to validate this association at higher resolution using mate-pair sequencing data. Because the epigenomes of breast cancer cells differ from those in human germline [30,31], we also asked if the pattern of structural mutability in breast cancer cells differ from the pattern of structural polymorphisms that arose in human germline. To answer these questions, we used three sets of maps of chromosomal breakpoints in breast cancer cell lines and tumors. The first breast cancer set was a map of aberrations we obtained from three breast cancer cell lines (HCC1954, MDA-MB-231, and MDA-MB-361) and two breast tumors. The tumor samples were obtained as anonymized samples from the National Breast Cancer Tissue Resource of NIH P50CA58183, maintained at the Baylor College of Medicine. All five samples were sequenced using SOLiD mate pair sequencing at a clone coverage of 9.7-13.5x. Breakpoints for all five samples were computed and analyzed using the toolset, and separated into germline and somatic enriched.

We imported additional breast cancer datasets from large studies [20,32] of breast tumors; the breakpoints were experimentally validated as being somatic. To ensure sufficient coverage of each of sample, we focused our analysis on samples that contained at least ten translocations.

**Figure 5:**
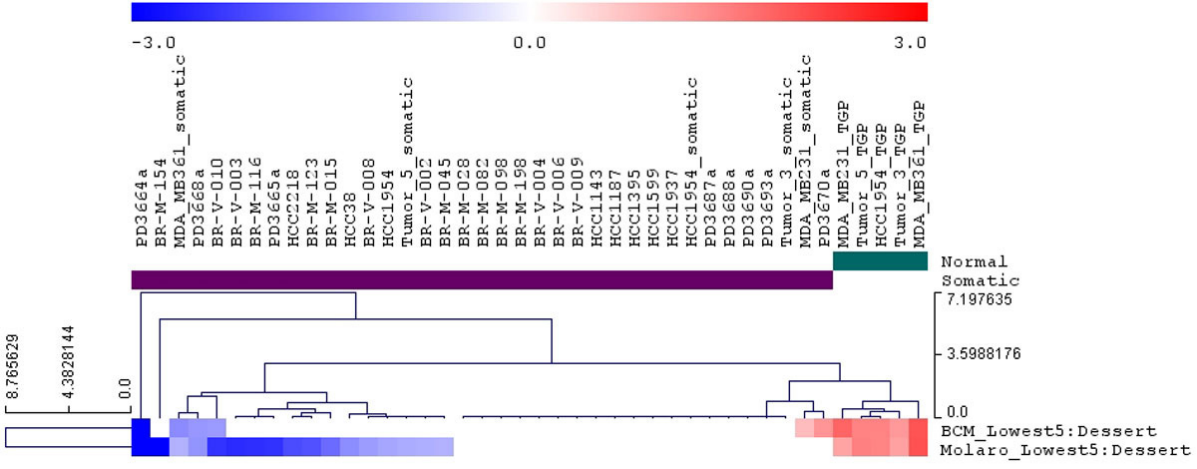
**Hierarchical clustering of breakpoint sets based on enrichment of germline methylation deserts**. DNA methylation deserts were defined as the 100 Kbp windows with the 5% lowest methylation in sperm. The two methylation desert tracks each corresponds to average methylation in two sperm samples, as described in [2]. Note the strong association of germline hypomethylation with breakpoints in germline but not with putatively somatic breakpoints.

We next imported from our previous study [2] the 5% subset of the 100 Kbp windows tiling the human genome that show the lowest methylation levels in human sperm samples (indicative of methylation levels in human germline). We applied the *Epigenomic Enrichment *tool to analyze the enrichment of these hypomethylated regions around breakpoints detected in breast cancer samples, including both germline breakpoints and putatively somatic ones. The results, summarized in Figure 5, show striking enrichment for hypomethylation around germline breakpoints and no enrichment around putatively somatic breakpoints.

#### Distinct patterns of epigenomic associations for structural mutability between germline and breast cancer

To further compare the pattern of structural mutability in breast cancer with the pattern of structural polymorphisms that arose in human germline, we analyzed enrichment scores for transcription factor binding sites and other epigenomic features around putative somatic and polymorphic breakpoints. We employed a total of 259 epigenomic feature tracks from the following sources: 148 transcription factor binding tracks generated by the ENCODE project [6,7]; 66 transcription factor binding tracks and 25 other epigenomic marks in breast cancer from the Cistrome database [33,34]; and 20 normal epigenomic tracks from the Human Epigenome Atlas Release 2 [8].

**Figure 6:**
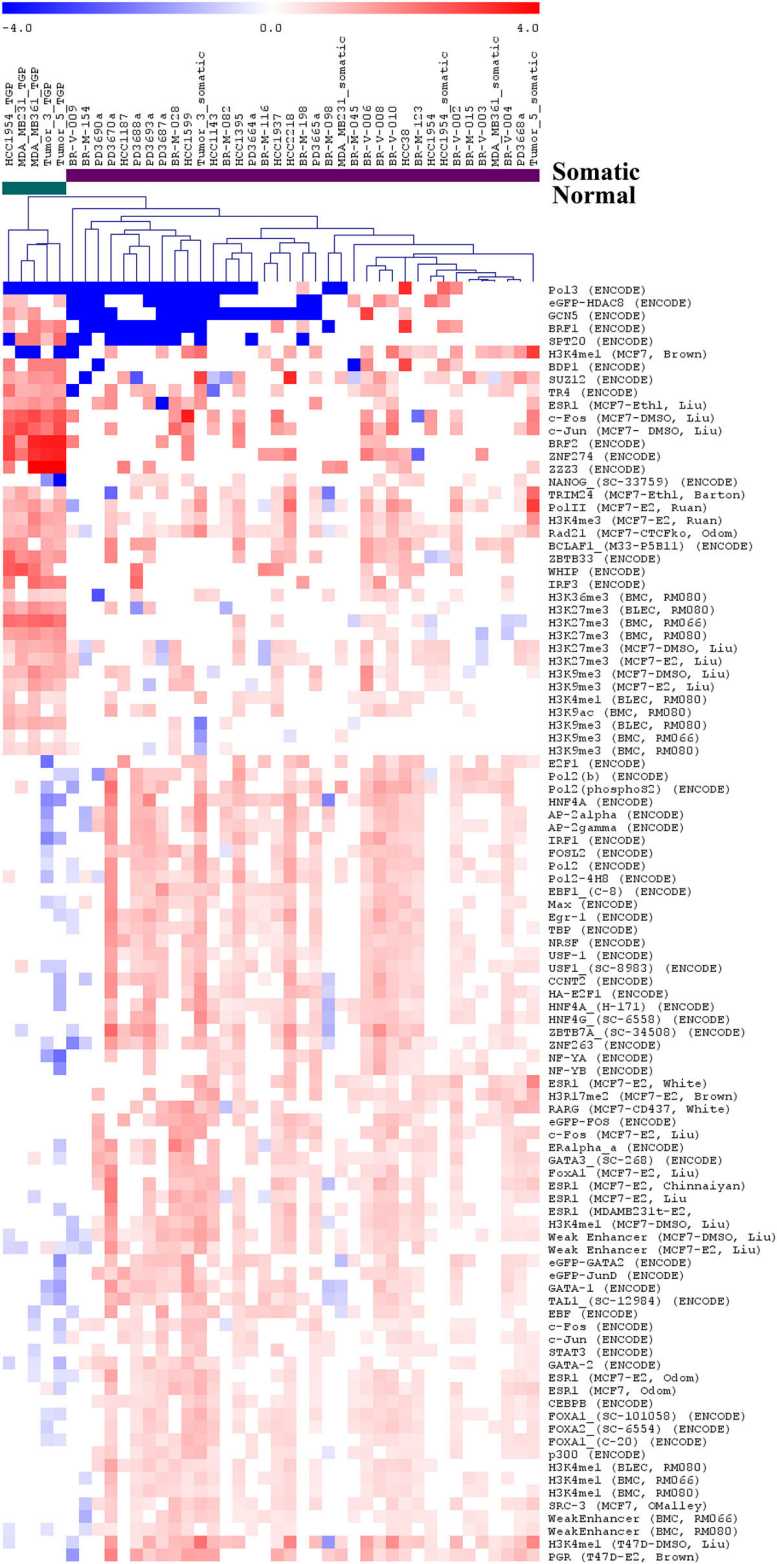
**Hierarchical clustering of breakpoint sets based on enrichment for transcription factor binding and epigenomic features**. Note the distinct patterns of enrichment for germline breakpoints (columns to the left) and putatively somatic breakpoints (columns to the right).

Using the *Epigenomic Enrichment *tool, we computed enrichment scores for each of the 259 epigenomic features for both germline and putatively somatic breakpoints using a 50,000 basepair radius around each breakpoint. A total of 107 (out of 259) features showed significantly different patterns of enrichment (Mann-Whitney-Wilcoxon test, p-value<0.05) between the two types of breakpoints. The enrichment scores for discriminating features were used to cluster the samples and the features (Figure 6). The clustering pattern suggests distinctly separate distributions of structural rearrangements in human germline and in breast cancer.

The enrichment score for 74 discriminating features was higher near somatic breakpoints, and for the remaining 33 discriminating epigenomic features it was higher near germline breakpoints. Among the 74 features enriched around somatic breakpoints were ESR1 (8 datasets, in 7 cases after treatment with estradiol), FoxA1 (2 datasets), PGR, and SRC-3. We observed strong enrichment around somatic translocations for 49 transcription factors profiled by the ENCODE project, including GATA-1/2, c-Jun, CEBPB, FoxA1, ERα, and p300. The enrichment patterns support previous results suggesting the role of ER in genomic instability. Specifically, we found strong and consistent enrichment for the estrogen receptor binding around somatic translocations. Patterns were similar for both ER-positive and ER-negative breast cancers, consistent with previous results [35-37]. To examine possible interactions between ESR1 and other transcription factors, for each of the ESR1 experiment, we identified somatic breakpoints within 50 kb of an ESR1 binding site, and then identified genes within 10 kb of such breakpoints. A total of 40 genes were identified by this method in least three cancer samples. Enrichment analysis of the 40 genes by GSEA indicated enrichment for ETS1 (p-value = 3.23 × 10^-4^) and p53 (p-value = 3.71 × 10^-4^) binding in promoter regions of these genes. The enrichment patterns suggest that transcription factors either individually or as part of larger complexes may promote genomic instability. Enrichment analysis around germline breakpoints (illustrated in Figure 6) revealed striking enrichment for binding sites of Suz12, a key member of the PRC2 Polycomb complex. This confirms our previous independent finding that PRC2 strongly associates with genomic instability in human germline [2]. Moreover, histone marks H3K27me3 and H3K9me3 show similar enrichment pattern, consistent with the established role of EZH2 and Suz12, the two constituent members of the PRC2 complex as respective "writers" of H3K27me3 and H3K9me3.

#### Somatic breakpoints associate with distal regulatory sites of active gene transcription

We examined enrichment patterns differences of the epigenomic marks from the Cistrome database mapped specifically in breast cancer cell lines. Within 50 Kbp of somatic breakpoints we found enrichment of H3K4me1, H3K9ac, and weak enhancers (defined according to [17]) and depletion of H3K27me3, H3K9me3, and H3K4me3. In summary, somatic translocations tend to preferentially occur at distal regulatory sites (enhancers) that carry open chromatin marks associated with active gene transcription, as opposed to promoters, gene bodies (actively transcribed or not), or areas with inactive chromatin marks.

We next examined genes regulated by mutable enhancers and, using gene set analysis, searched for other regulators that may be indirectly associated with genomic instability. For each of the enriched enhancer datasets, we identified somatic breakpoints within 50 kb of an enhancer site, then the genes within 10 kb of such translocations. After limiting the genes to those nominated in at least 2 somatic samples, we obtained a list of 127 individual genes, including known oncogenes such as ERBB2, IGF1R, and MYC. Using the ENCODE dataset, we found enrichments for p300 binding in the vicinity of somatic translocations. Gene ontology and pathway analysis (GSEA) [26,27] revealed a significant enrichment in genes with promoter regions containing motifs bound by *MEIS1 *(p-value = 8.12 × 10^-9^.), *MYOD1 *(p-value = 5.18 × 10^-6^), *ETS2 *(p-value = 4.57 × 10^-5^), and *P53 *(p-value = 9.17 × 10^-4^).

#### Distinct patterns of structural mutability in breast cancer cell lines and tumors

**Figure 7:**
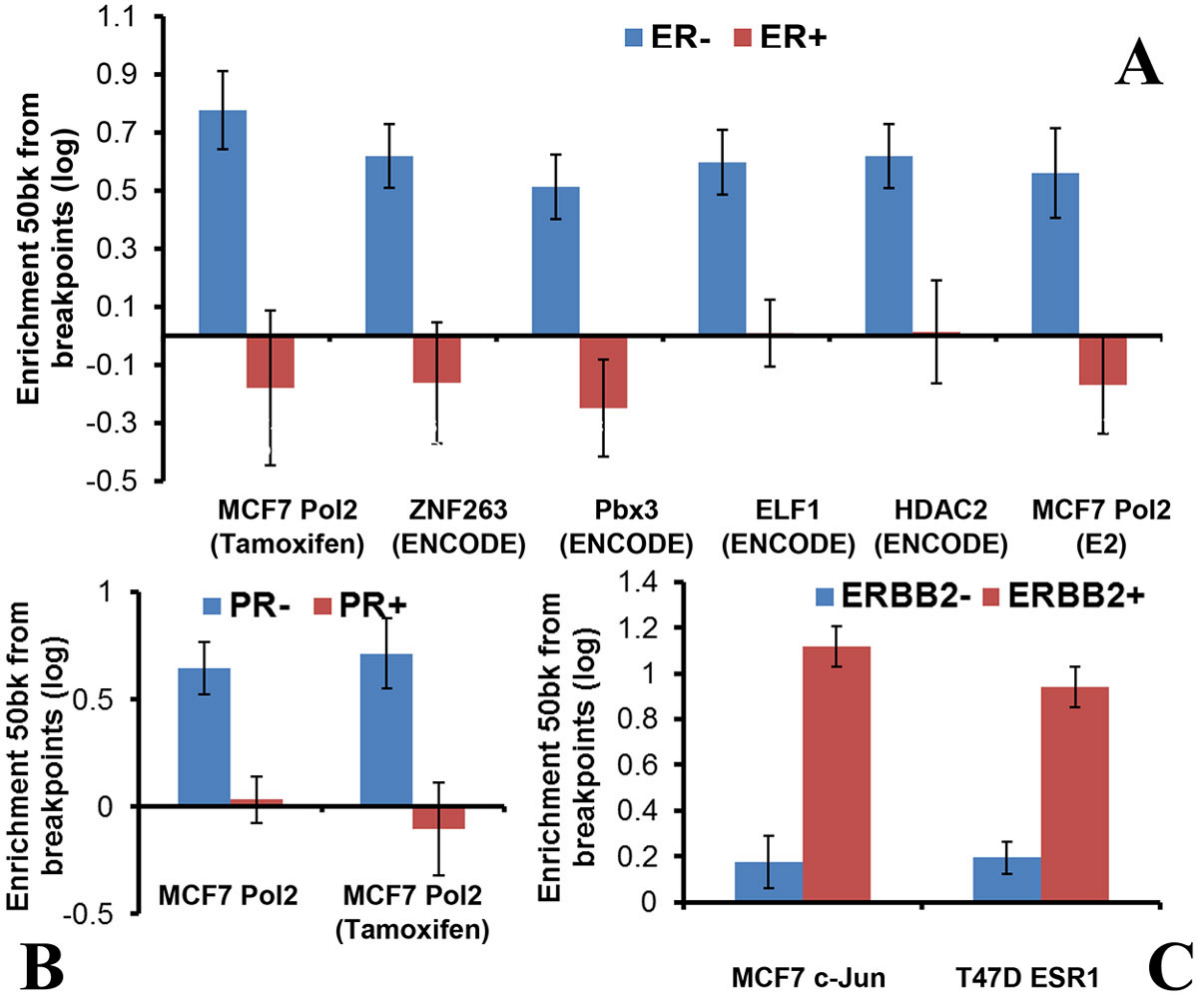
**Patterns of enrichment segregating breast cancer subtypes by estrogen receptor status (ER), progesterone receptor status (PR), or HER2 status (ERBB2)**. (A) ER+ vs ER-; note that genes with the Pol2 mark and transcribed in MCF7, an ER+ cell line, are affected by breakpoints in ER- cells but not in ER+ cells. (B) PR+ vs PR-. (C) HER2+ vs HER2-.

Breast cancer subtypes are defined using the expression levels of key genes such as estrogen receptor (ER), progesterone receptor (PR), and erbB-2 (HER-2/neu). We explored breakpoint patterns in cancer cell lines and tumors annotated for ER, PR, or HER2 status. As before, the breakpoint patterns were characterized by enrichment of nearby transcription factor binding sites and epigenomic features. We determined distinct pattern of enrichments (t-test, p < 0.05, enrichment fold change>1.25x) for each of the three genes. ER- cell lines and tumors enrich for RNA polymerase II (two datasets determined in MCF7 cells), and the ENCODE ChIP-Seq datasets of ZNF263, Pbx3, ELF1, and HDAC2. This might indicate that genes transcribed in MCF7, an ER+ cell line, are affected by breakpoints significantly more in ER- cells compared to ER+ cells. PR- cell lines and tumors enrich for RNA Polymerase II (two MCF7 datasets, either untreated or treated with estradiol and tamoxifen). Since in many cells ER and PR levels are correlated, genes transcribed in MCF7 have higher association with breakpoints in PR- cells compared to PR+ cells. Finally, HER2+ cell lines and tumors enrich for c-Jun and ESR1.A summary of the distinct enrichment patterns is presented in Figure 7.

## Discussion

The challenges of identifying, integrating and interpreting chromosomal aberrations in cancer in the context of the epigenome can be effectively addressed using the Breakout algorithm and related tools within the Genboree Workbench. Researchers are empowered to translate the results of large genome-wide experiments into meaningful and experimentally testable hypotheses. The Genboree Workbench framework allows integration of other breakpoint callers, visualization packages, and additional integrative analysis tools.

By applying Breakout and other Genboree Workbench tools we mapped breakpoints in breast and prostate cancer cell lines and tumors, discriminated between polymorphic breakpoints of germline origin and those of somatic origin, and analyze both types of breakpoints in the context of the Human Epigenome Atlas, ENCODE and Cistrome databases. Using the toolset we identified somatic structural variants in prostate cancer, genes affected by the variants, and detected epigenomic footprints of their regulation.

We confirm and extend our previous findings about the association of the epigenome and selective structural mutability of the human genome [2]. We validate the association between hypomethylation and genomic instability in germline at higher resolution using independent mate-pair sequencing data from the 1000 Genomes Project. We also show that only germline breakpoints show striking enrichment for regions hypomethylated in germline but that somatic breakpoints detected in breast cancer do not. The original study established association between breakpoints in human germline and the binding sites of the PRC2 polycomb complex only indirectly. We now validate this association directly by performing an extensive meta-analysis using ChIP-seq data and epigenomic marks from ENCODE, Cistrome and other projects. As anticipated by the previously reported results, the breakpoints in germline strongly associate with binding sites of Suz12, a key member of the PRC2 Polycomb complex, and with PRC2-associated histone marks H3K27me3 and H3K9me3. The breakpoints in breast cancer associate with different sets of transcription factor binding sites and epigenomic states, such as distal regulatory sites associated with active gene transcription. Finally, we identify distinct patterns of selective structural mutability in breast cancer cell lines that associate with the status of key oncogenes such as ER, PR, or HER2.

In summary, the results obtained using Breakout and related tools in the Genboree Workbench suggest that structural mutations are not randomly distributed relative to the epigenome. Cell-type specific patterns of associations between epigenomic states and structural mutations suggest that the epigenome and transcription factors play roles in determining selective structural mutability of the genome in both somatic cells and in germline.

### Software availability and requirements

Breakout and the other tools presented are part of the Genboree Workbench and can be accessed at the address http://genboree.org/java-bin/workbench.jsp. Supported browsers are Internet Explorer versions 8 and above, Mozilla Firefox versions 7 and above. A tutorial and sample datasets are available at http://genboree.org/breakpoints. Breakout and the epigenome enrichment analysis tools can be downloaded at http://www.brl.bcm.tmc.edu/breakout/breakoutDownload.rhtml.

## Competing interests

AM founded and owns shares in IP Genesis, Inc., a corporation which owns an exclusive license from Baylor College of Medicine for commercial use of the Genboree trademark. The other authors have no competing interests.

## Authors' contributions

CC, CSP, ARJ, SEM, and AM conceived the Structural Variation Analysis Toolset and co-wrote the manuscript. CC and AM conceived and implemented Breakout. AM and ARJ conceived the Genboree Workbench and Genboree REST APIs. ARJ, SP, AT, and SR implemented the Genboree Workbench and integrated the structural variation analysis tools via the Genboree REST APIs. CC, CSP, VA, AVL, and SEM performed data analysis. AM supervised the Genboree project.

## Declaration

Funding for the publication of this article was provided by the Caroline Wiess Law Foundation [to SEM].

This article has been published as part of *BMC Bioinformatics *Volume 15 Supplement 7, 2014: Selected articles from the 10th Annual Biotechnology and Bioinformatics Symposium (BIOT 2013). The full contents of the supplement are available online at http://www.biomedcentral.com/bmcbioinformatics/supplements/15/S7

## References

[B1] Muller HJ The remaking of chromosomes Collecting Net 1938 XIII 181 195

[B2] Li J Harris RA Cheung SW Coarfa C Jeong M Goodell MA White LD Patel A Kang SH Shaw C Genomic hypomethylation in the human germline associates with selective structural mutability in the human genome PLoS genetics 2012 8 e1002692 10.1371/journal.pgen.1002692 22615578 PMC3355074

[B3] Siva N 1000 Genomes project Nat Biotechnol 2008 26 256 18327223 10.1038/nbt0308-256b

[B4] Mills RE Walter K Stewart C Handsaker RE Chen K Alkan C Abyzov A Yoon SC Ye K Cheetham RK Mapping copy number variation by population-scale genome sequencing Nature 2011 470 59 65 10.1038/nature09708 21293372 PMC3077050

[B5] A map of human genome variation from population-scale sequencing Nature 2010 467 1061 1073 10.1038/nature09534 20981092 PMC3042601

[B6] Birney E Stamatoyannopoulos JA Dutta A Guigo R Gingeras TR Margulies EH Weng Z Snyder M Dermitzakis ET Thurman RE Identification and analysis of functional elements in 1% of the human genome by the ENCODE pilot project Nature 2007 447 799 816 10.1038/nature05874 17571346 PMC2212820

[B7] Myers RM Stamatoyannopoulos J Snyder M Dunham I Hardison RC Bernstein BE Gingeras TR Kent WJ Birney E Wold B Crawford GE A user's guide to the encyclopedia of DNA elements (ENCODE) PLoS Biol 2011 9 e1001046 10.1371/journal.pbio.1001046 21526222 PMC3079585

[B8] Bernstein BE Stamatoyannopoulos JA Costello JF Ren B Milosavljevic A Meissner A Kellis M Marra MA Beaudet AL Ecker JR The NIH Roadmap Epigenomics Mapping Consortium Nature biotechnology 2010 28 1045 1048 10.1038/nbt1010-1045 20944595 PMC3607281

[B9] Li H Handsaker B Wysoker A Fennell T Ruan J Homer N Marth G Abecasis G Durbin R The Sequence Alignment/Map format and SAMtools Bioinformatics 2009 25 2078 2079 10.1093/bioinformatics/btp352 19505943 PMC2723002

[B10] Homer N Merriman B Nelson SF BFAST: an alignment tool for large scale genome resequencing PLoS One 2009 4 e7767 10.1371/journal.pone.0007767 19907642 PMC2770639

[B11] Li H Durbin R Fast and accurate long-read alignment with Burrows-Wheeler transform Bioinformatics 2010 26 589 595 10.1093/bioinformatics/btp698 20080505 PMC2828108

[B12] Li H Durbin R Fast and accurate short read alignment with Burrows-Wheeler transform Bioinformatics 2009 25 1754 1760 10.1093/bioinformatics/btp324 19451168 PMC2705234

[B13] Coarfa C Yu F Miller CA Chen Z Harris RA Milosavljevic A Pash 3.0: A versatile software package for read mapping and integrative analysis of genomic and epigenomic variation using massively parallel DNA sequencing BMC Bioinformatics 2010 11 572 10.1186/1471-2105-11-572 21092284 PMC3001746

[B14] Lister R Pelizzola M Dowen RH Hawkins RD Hon G Tonti-Filippini J Nery JR Lee L Ye Z Ngo QM Human DNA methylomes at base resolution show widespread epigenomic differences Nature 2009 462 315 322 10.1038/nature08514 19829295 PMC2857523

[B15] Harris RA Wang T Coarfa C Nagarajan RP Hong C Downey SL Johnson BE Fouse SD Delaney A Zhao Y Comparison of sequencing-based methods to profile DNA methylation and identification of monoallelic epigenetic modifications Nat Biotechnol 2010 28 1097 1105 10.1038/nbt.1682 20852635 PMC2955169

[B16] Lister R Pelizzola M Kida YS Hawkins RD Nery JR Hon G Antosiewicz-Bourget J O'Malley R Castanon R Klugman S Hotspots of aberrant epigenomic reprogramming in human induced pluripotent stem cells Nature 2011 471 68 73 10.1038/nature09798 21289626 PMC3100360

[B17] Ernst J Kheradpour P Mikkelsen TS Shoresh N Ward LD Epstein CB Zhang X Wang L Issner R Coyne M Mapping and analysis of chromatin state dynamics in nine human cell types Nature 2011 473 43 49 10.1038/nature09906 21441907 PMC3088773

[B18] Maunakea AK Nagarajan RP Bilenky M Ballinger TJ D'Souza C Fouse SD Johnson BE Hong C Nielsen C Zhao Y Conserved role of intragenic DNA methylation in regulating alternative promoters Nature 2010 466 253 257 10.1038/nature09165 20613842 PMC3998662

[B19] Richardson L Ruby S RESTful web services 2007 Sebastopol, Calif.: O'Reilly

[B20] Stephens PJ McBride DJ Lin ML Varela I Pleasance ED Simpson JT Stebbings LA Leroy C Edkins S Mudie LJ Complex landscapes of somatic rearrangement in human breast cancer genomes Nature 2009 462 1005 1010 10.1038/nature08645 20033038 PMC3398135

[B21] Hormozdiari F Alkan C Eichler EE Sahinalp SC Combinatorial algorithms for structural variation detection in high-throughput sequenced genomes Genome Res 2009 19 1270 1278 10.1101/gr.088633.108 19447966 PMC2704429

[B22] Sindi S Helman E Bashir A Raphael BJ A geometric approach for classification and comparison of structural variants Bioinformatics 2009 25 i222 230 10.1093/bioinformatics/btp208 19477992 PMC2687962

[B23] Hormozdiari F Hajirasouliha I Dao P Hach F Yorukoglu D Alkan C Eichler EE Sahinalp SC Next-generation VariationHunter: combinatorial algorithms for transposon insertion discovery Bioinformatics 2010 26 i350 357 10.1093/bioinformatics/btq216 20529927 PMC2881400

[B24] Hampton OA Den Hollander P Miller CA Delgado DA Li J Coarfa C Harris RA Richards S Scherer SE Muzny DM A sequence-level map of chromosomal breakpoints in the MCF7 breast cancer cell line yields insights into the evolution of a cancer genome Genome Res 2009 19 167 177 19056696 10.1101/gr.080259.108 PMC2652200

[B25] Futreal PA Coin L Marshall M Down T Hubbard T Wooster R Rahman N Stratton MR A census of human cancer genes Nature reviews Cancer 2004 4 177 183 10.1038/nrc1299 14993899 PMC2665285

[B26] Subramanian A Tamayo P Mootha VK Mukherjee S Ebert BL Gillette MA Paulovich A Pomeroy SL Golub TR Lander ES Mesirov JP Gene set enrichment analysis: a knowledge-based approach for interpreting genome-wide expression profiles Proc Natl Acad Sci USA 2005 102 15545 15550 10.1073/pnas.0506580102 16199517 PMC1239896

[B27] Mootha VK Lindgren CM Eriksson KF Subramanian A Sihag S Lehar J Puigserver P Carlsson E Ridderstrale M Laurila E PGC-1alpha-responsive genes involved in oxidative phosphorylation are coordinately downregulated in human diabetes Nat Genet 2003 34 267 273 10.1038/ng1180 12808457

[B28] Kastner P Krust A Turcotte B Stropp U Tora L Gronemeyer H Chambon P Two distinct estrogen-regulated promoters generate transcripts encoding the two functionally different human progesterone receptor forms A and B EMBO J 1990 9 1603 1614 2328727 10.1002/j.1460-2075.1990.tb08280.x PMC551856

[B29] Yu J Mani RS Cao Q Brenner CJ Cao X Wang X Wu L Li J Hu M Gong Y An integrated network of androgen receptor, polycomb, and TMPRSS2-ERG gene fusions in prostate cancer progression Cancer Cell 2010 17 443 454 10.1016/j.ccr.2010.03.018 20478527 PMC2874722

[B30] Radpour R Kohler C Haghighi MM Fan AX Holzgreve W Zhong XY Methylation profiles of 22 candidate genes in breast cancer using high-throughput MALDI-TOF mass array Oncogene 2009 28 2969 2978 10.1038/onc.2009.149 19503099

[B31] Dedeurwaerder S Defrance M Calonne E Denis H Sotiriou C Fuks F Evaluation of the Infinium Methylation 450K technology Epigenomics 2011 3 771 784 10.2217/epi.11.105 22126295

[B32] Banerji S Cibulskis K Rangel-Escareno C Brown KK Carter SL Frederick AM Lawrence MS Sivachenko AY Sougnez C Zou L Sequence analysis of mutations and translocations across breast cancer subtypes Nature 2012 486 405 409 10.1038/nature11154 22722202 PMC4148686

[B33] Liu T Ortiz JA Taing L Meyer CA Lee B Zhang Y Shin H Wong SS Ma J Lei Y Cistrome: an integrative platform for transcriptional regulation studies Genome biology 2011 12 R83 10.1186/gb-2011-12-8-r83 21859476 PMC3245621

[B34] Qin B Zhou M Ge Y Taing L Liu T Wang Q Wang S Chen J Shen L Duan X CistromeMap: A knowledgebase and web server for ChIP-Seq and DNase-Seq studies in mouse and human Bioinformatics 2012 10.1093/bioinformatics/bts157 PMC3348563 22495751

[B35] Fang M Toher J Morgan M Davison J Tannenbaum S Claffey K Genomic differences between estrogen receptor (ER)-positive and ER-negative human breast carcinoma identified by single nucleotide polymorphism array comparative genome hybridization analysis Cancer 2011 117 2024 2034 10.1002/cncr.25770 21523713 PMC4521590

[B36] Kabil A Silva E Kortenkamp A Estrogens and genomic instability in human breast cancer cells--involvement of Src/Raf/Erk signaling in micronucleus formation by estrogenic chemicals Carcinogenesis 2008 29 1862 1868 10.1093/carcin/bgn138 18544561

[B37] Melchor L Honrado E Huang J Alvarez S Naylor TL Garcia MJ Osorio A Blesa D Stratton MR Weber BL Estrogen receptor status could modulate the genomic pattern in familial and sporadic breast cancer Clinical cancer research : an official journal of the American Association for Cancer Research 2007 13 7305 7313 10.1158/1078-0432.CCR-07-0711 18094411

